# Anterior cingulate cortex and insula response during indirect and direct processing of emotional faces in generalized social anxiety disorder

**DOI:** 10.1186/2045-5380-3-7

**Published:** 2013-04-02

**Authors:** Heide Klumpp, David Post, Mike Angstadt, Daniel A Fitzgerald, K Luan Phan

**Affiliations:** 1 Mood and Anxiety Disorders Research Program, Department of Psychiatry (HK, DP, DAF, KLP), University of Illinois at Chicago, 1747 W. Roosevelt Rd, Chicago, IL, 60608, USA; 2 Department of Psychiatry (MA, KLP), University of Michigan, Ann Arbor, MI, USA; 3 Neuropsychiatric Research Program (KLP), Mental Health Service, Jesse Brown VA Medical Center, Chicago, IL, USA

**Keywords:** Social anxiety, fMRI, Emotional faces, Threat processing, Brain imaging

## Abstract

**Background:**

Generalized social anxiety disorder (gSAD) is associated with a heightened neural sensitivity to signals that convey threat, as evidenced by exaggerated amygdala and/or insula activation when processing face stimuli that express negative emotions. Less clear in the brain pathophysiology of gSAD are cortical top down control mechanisms that moderate reactivity in these subcortical emotion processing regions. This study evaluated amygdala, insula, and anterior cingulate cortex (ACC) activity in gSAD with a novel “Emotional Faces Shifting Attention Task” (EFSAT), an adaptation of perceptual assessment tasks well-known to elicit amygdala response. In healthy volunteers, the task has been shown to engage the amygdala when attention is directed to emotional faces and the ACC when attention is directed to shapes, away from emotional faces.

**Methods:**

During functional MRI, 29 participants with gSAD and 27 healthy controls viewed images comprising a trio of faces (angry, fear, or happy) alongside a trio of geometric shapes (circles, rectangles, or triangles) within the same field of view. Participants were instructed to match faces or match shapes, effectively directing attention towards or away from emotional information, respectively.

**Results:**

Participants with gSAD exhibited greater insula, but not amygdala, activation compared to controls when attending to emotional faces. In contrast, when attention was directed away from faces, controls exhibited ACC recruitment, which was not evident in gSAD. Across participants, greater ACC activation was associated with less insula activation.

**Conclusions:**

Evidence that individuals with gSAD exhibited exaggerated insula reactivity when attending to emotional faces in EFSAT is consistent with other studies suggesting that the neural basis of gSAD may involve insula hyper-reactivity. Furthermore, greater ACC response in controls than gSAD when sustained goal-directed attention is required to shift attention away from social signals, together with a negative relationship between ACC and bilateral insula activity, indicate the ACC may have served a regulatory role when the focus of attention was directed to shapes amidst emotional faces.

## Background

Generalized social anxiety disorder (gSAD) is characterized by extreme fears of potential scrutiny encompassing most social situations [1,2] and, therefore, can lead to impaired functioning in educational, occupational, and interpersonal domains [1,3,4]. Clinical manifestations of gSAD reflect a heightened threat processing system. These include excessive attention to negative social-signals (e.g., angry, fearful facial expressions [5]); fear-based physiological responses (autonomic changes) in anticipation of [6,7], or during [8], anxiety-evoking situations; and negative predictions about social events [9].

Thus far, models of the neural pathophysiology of gSAD have focused on an enhanced threat processing system that have fundamentally evolved from amygdala-centric ‘functional’ brain activation paradigms. In light of the central role amygdala plays in mediating fear and threat-related processing [7,10-12], its response to emotional information has been a predominant focus in affective neuroscience. As one example, when coupled with functional neuroimaging, perceptual matching tasks are designed to isolate the influence of emotional face content by contrasting a matching face condition with a sensorimotor control condition (i.e., matching shapes) and to robustly elicit amygdala response in healthy volunteers [13-16]. Building on this paradigm, studies of gSAD have shown amygdala reactivity to threat exceeds that of healthy individuals [17,18] and the extent of this reactivity has been shown to reflect symptom severity [19].

There is increasing evidence that the neural substrates of gSAD extend beyond the amygdala. In addition, there is a growing realization that functional neuroimaging paradigms should move beyond perceptual assessment tasks that primarily probe subcortical reactivity in key emotion processing regions. First, accumulating data point to insula hyper-reactivity in regard to processing negative emotional information in gSAD [20-27], and evidence of a correlation between anterior insula (aINS) reactivity to threat-relevant cues and symptom severity [24] also supports the notion that exaggerated aINS reactivity underlies anxiety disorders [28,29]. It has been posited that negative beliefs are mediated by feeling states, a core function of the aINS [30,31]. That is, aberrant insula activiation in anxiety is thought to be driven by sensitivity to aversive interoceptive signals and/or inaccurate interpretations of ordinary changes in bodily state [28,29]. Second, the prefrontal cortex (PFC) has reciprocal connections with the amygdala [32-34] and aINS [30,35-39], yet less is known about prefrontal mechanisms in gSAD. The relative gap in knowledge may relate to the interest in aberrant amygdala activation in anxiety and use of perceptual matching tasks, which are not well-validated to probe PFC regions.

Several functional neuroimaging tasks exist that are known to robustly recruit PFC areas by engaging cognitive functions such as ‘top down’ attentional control—that is, the ability to effectively resolve the type of conflict that occurs when cognitive goals compete with salient distractors for limited processing resources [40-48]. For example, to reflect the competition for processing resources imposed by distractors, tasks such as the emotional counting Stroop [48-50], modified dot probe detection [51,52], and “faces/houses” [40,53] have in common the rapid and simultaneous presentation of non-emotional, task-relevant stimuli and salient distractors (e.g., 500 ms or less; [40,51-54]). Studies using such tasks have shown that anxiety-prone [40,41,55,56] and clinically anxious patients [49,50,57], including those with gSAD [49], exhibit deficiencies in the recruitment of the anterior cingulate cortex (ACC) and other emotion regulation areas (e.g., dorsolateral PFC) in the presence of threat distractors. Excessive attention to threat appears to encompass impoverished top-down control at least when the window of information processing is markedly restricted (i.e., when visual processing is fast).

Current models propose that there is a balance between attending to the task at hand and to the emotional salience that surround the given task. Resolving emotional conflict must occur in the context that salient emotional cues not only capture but sustain visual attention [58]. Consequently, prefrontal areas should engage during the maintenance of goal-directed attention even when the window of information processing is extended to that of direct emotion processing.

**Figure 1:**
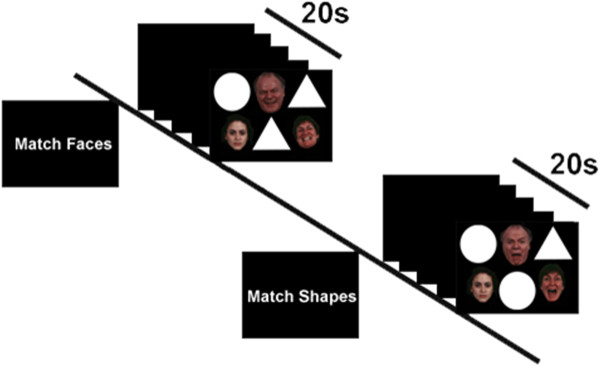
Schematic of an exemplar Match Faces and Match Shapes blocks in the Emotional Faces Shifting Attention Task (EFSAT).

In order to capture this balance, we adapted the face matching task into a relatively slow, easy-to-perform blocked perceptual matching task known as the “Emotional Faces Shifting Attention Task” (EFSAT), which requires subjects to shift their attention towards and away from emotional faces. In contrast to the traditional faces-only and shapes-only images, in EFSAT both image types were configured to be in the same field of view (Figure 1). Therefore, the instruction to “Match Shapes” directed attention *away* from emotional faces whereas “Match Faces” directed attention *towards* emotional faces. Analogous to traditional perceptual assessment tasks, each matching trial was presented for 4 sec in back-to-back blocks. In a recent study [59], we demonstrated the modification was successful as healthy volunteers engaged the amygdala to “Match Faces” and the rostral ACC to “Match Shapes” indicating the ACC effectively impeded the processing of task-irrelevant emotional faces presented alongside shapes. Thus, EFSAT complements traditional attentional control paradigms shown to recruit PFC areas.

In an effort to assess neural correlates of sustained attentional control in gSAD, participants performed the EFSAT during functional MRI. Our predictions for emotion processing (Match Faces > Match Shapes) were individuals with gSAD compared to demographically matched healthy controls (HC) would demonstrate: 1) exaggerated amygdala and anterior insula reactivity, 2) a positive relationship between activation in these regions and symptom severity, and 3) subcortical effects would be most pronounced when the faces constituted social signals of threat (angry and fear). Regarding attentional control (Match Shapes > Match Faces), we predicted gSAD compared to HC would exhibit: 1) reduced ACC activation, and 2) deficient ACC response would be most pronounced in the presence of distracting threat faces. Lastly, given reciprocal subcortical-ACC associations, we hypothesized insula activation to emotion processing would negatively correlate with ACC response during attentional control across participants.

## Methods

### Participants

Twenty-seven matched HC were recruited through community advertisements. The current HC group incorporates six new participants to the cohort in the previous EFSAT study involving only healthy volunteers [59], which previously served as a validation of this novel task and prompted pursuing the current study and informed the stated hypotheses. Twenty-nine individuals diagnosed with gSAD were identified through community advertisement and an outpatient psychiatric clinic. Participants completed the Structured Clinical Interview for DSM-IV [60] conducted by a licensed psychologist in addition to measures of symptoms and negative mood, such as the Liebowitz Social Anxiety Scale “LSAS” [61] and Beck Depression Inventory “BDI” [62]. All of the subjects were free of psychotropic medications except for two individuals with gSAD who were taking a selective serotonin reuptake inhibitor. None of the participants with gSAD had a current major depressive episode or severe depression symptoms (i.e., BDI score of 30 or greater; [62]). Furthermore, none of the participants with gSAD had recent substance abuse/dependence (within 6 months of study) or a lifetime history of major psychiatric illness (e.g., bipolar, psychotic disorder). All participants were between 18 and 55 years of age, right-handed, and free of current and past major medical or neurologic illness, as confirmed by a Board Certified physician. None of the participants tested positive for alcohol or illegal substances. GSAD and HC groups were matched for age (*t*(54) = 0.02, *p* = 0.99), gender (χ^2^(1, N = 56) = 0.25, *p* = 0.62), and race/ethnicity (χ^2^(3, N = 56) = 4.54, *p* = 0.21). All participants provided written informed consent, as approved by the Institutional Review Boards (ethics committee) of the University of Michigan Medical School in compliance with the Helsinki Declaration. See Table 1 for group characteristics.

**Table 1:** Group characteristics (Mean ± SD)

	**gSAD (n = 29)**	**HC (n = 27)**	**t (*df*** **= 56)**	***p***
Age (years)	24.9 ± 6.3	24.9 ± 5.9	0.02	0.99
Gender	18 F/11 M	15 F/12 M		
Race/Ethnicity	6Asa/3AA/2H/18C	3Asa/1AA/0H/23C		
Social Anxiety Severity	77.3 ± 15.4	7.8 ± 6.3	21.69	<0.001
State Anxiety Level	42.9 ± 8.6	24.0 ± 5.4	9.72	<0.001
Trait Anxiety Level	49.1 ± 8.4	26.6 ± 4.0	12.69	<0.001
Depression Level	12.0 ± 7.9	0.67 ± 1.0	7.41	<0.001

gSAD, Generalized Social Anxiety Disorder participants.

HC, Healthy Control participants.

F, Female.

M, Male.

Asa, Asian American; AA, African American; H, Hispanic; C, Caucasian.

Social anxiety severity measured with the Liebowitz Social Anxiety Scale.

State and trait anxiety levels measured with the Spielberger State-Trait Anxiety Inventory.

Depression level measured with the Beck Depression Inventory.

### Emotional faces shifting attention task

During fMRI scanning, participants performed the EFSAT, which consisted of trials depicting a trio of geometric shapes (circles, rectangles, triangles) presented alongside a trio of faces within the same field of view (Figure 1). During the “Match Faces” condition, participants had to select from one of two bottom faces (one emotional vs. one neutral) which matched the emotional expression of the top target face, whereas in the “Match Shapes” condition, participants selected from the two top shapes the one matching the bottom target shape. The faces were chosen from a validated stimulus set [63], were presented without repetition, and equally represented both genders.

The task comprised 36 back-to-back blocks: 18 blocks of matching shapes interleaved with 18 blocks of matching emotional faces, counterbalanced across two runs. Each target emotional face condition (angry, fear, happy) was presented in six 20-second blocks; these were presented pseudo-randomly, without subsequent repetition of individual faces. Each block began with a 4 second cue to either “Match Faces” (attend *to* faces) or “Match Shapes” (attend *away* from faces) followed by the four sequential matching trials, each lasting 4 seconds. Participant responses were recorded via button press.

### Functional imaging: acquisition and analysis

Functional imaging was performed with blood-oxygen-level-dependent (BOLD) sensitive whole-brain fMRI on a 3.0 Tesla GE Signa System (General Electric; Milwaukee, WI) using a standard radio frequency coil. Images were acquired with 30 axial, 5-mm-thick slices using a standard T2*-sensitive gradient echo reverse spiral acquisition sequence (2 s repetition time; 25 ms echo time; 64 × 64 matrix; 24 cm field of view; flip angle 77°; 3.75 × 3.75 × 5 mm final voxel size). A high-resolution, T1-weighted volumetric anatomical scan was also acquired for anatomical localization. Data from all participants met the criteria for quality with minimal motion correction (movements were less than 2 mm in any one direction across each functional run) and the first 4 volumes from each run were discarded to allow for T1 equilibration effects. Conventional preprocessing steps were used in Statistical Parametric Mapping (SPM8) software package (Wellcome Trust Centre for Neuroimaging, London http://www.fil.ion.ucl.ac.uk/spm). Briefly, images were temporally corrected to account for differences in slice time collection, spatially realigned to the first image of the first run, normalized to a Montreal Neurological Institute (MNI) template, and smoothed with an 8 mm isotropic Gaussian kernel.

A general linear model was applied to the time series, convolved with the canonical hemodynamic response function and with a 128 s high-pass filter. Blocks of Match Faces (shapes in ‘background’) and Match Shapes (faces in ‘background’) were modeled separately based on the target emotion or shape (angry, fearful, or happy / circle, square, or triangle) resulting in six regressors, the effects of which were estimated for each voxel for each participant and taken to the second level for random effects analysis.

A region of interest (ROI)-based Analysis of Variance (ANOVA) was conducted to test our *a priori* hypotheses—specifically, within the bilateral amygdala, anterior insula, and anterior cingulate cortex (ACC) as defined by atlas-based anatomical ROIs with Automated Anatomical Labeling (AAL) toolbox within SPM, in the context of main effects of Group (gSAD, HC), Emotion (angry, fear, happy), and Group by Emotion interaction. Activations were deemed significant at a p-value of <0.05, corrected for multiple comparisons within the *a priori* anatomically-confined ROI (i.e., a small volume correction [SVC]). For completeness, we also report activation clusters outside *a priori* ROIs at a whole-brain threshold of p < 0.001 uncorrected with at least 10 contiguous voxels.

To clarify the direction of activation and to examine correlations, parameter estimates of peak activation (β weights, arbitrary units [a.u.]) were extracted from spherical (10-mm diameter) ROIs from each participant and submitted to *post hoc* t-tests in the Statistical Package for the Social Sciences (SPSS) (Chicago, IL version 18).

Pearson correlational analyses were conducted to evaluate the predicted positive relationship between amygdala and/or insula activation with symptom severity, and the association between subcortical (amygdala, aINS) activation and ACC response.

Behavioral data were submitted to a 2 (Group: gSAD, HC) x 3 (Emotion: fear, angry, happy) ANOVA with repeated measures for the Emotion factor for Match Faces and emotional ‘distractor’ for Match Shapes. All significant main effects and interactions were followed up with two-tailed t-tests, p < 0.05.

## Results

### Behavioral data: match faces

For accuracy, results revealed a main effect of Emotion [*F*(2,108) 4.41, *p* < 0.016] but no main effect of Group or Emotion x Group interaction (all *p*_*s*_ > 0.05). The main effect of Emotion showed participants were more accurate at matching fearful than angry faces (p < 0.007); no differences were evident for fearful versus happy (p = 0.13) or angry versus happy faces (p = 0.12). Similarly, mean reaction times (RT) for accurate trials showed a main effect of Emotion [*F*(2,108) 36.0, *p* < 0.001] but no main effect of Group or Emotion x Group interaction (all *p*_*s*_ > 0.05). The main effect of Emotion revealed participants were faster at matching happy than angry faces (*p* < 0.001) and faster at matching fearful than angry faces (*p* < 0.001). There was a non-significant trend for matching fearful faster than happy faces (*p* = 0.08). See Table 2 for behavioral descriptives.

**Table 2:** Accuracy and reaction times (in milliseconds) for accurate trials (Mean ± SD)

**Contrast**	**gSAD (n = 29)**	**HC (n = 27)**	**t (*df*** **= 56)**	***p***
Accuracy				
Attend Angry vs. Shapes	85.6 ± 11.8	86.6 ± 10.9	0.31	0.76
Attend Fear vs. Shapes	92.7 ± 9.1	90.7 ± 10.1	0.75	0.46
Attend Happy vs. Shapes	90.8 ± 11.0	86.3 ± 14.2	1.34	0.19
Attend Shapes vs. Angry (distractor)	94.1 ± 8.1	93.7 ± 8.0	0.20	0.84
Attend Shapes vs. Fear (distractor)	90.1 ± 14.4	95.5 ± 7.6	1.75	0.09
Attend Shapes vs. Happy (distractor)	91.2 ± 11.5	93.4 ± 7.5	0.81	0.42
Reaction Times				
Attend Angry vs. Shapes	1479.6 ± 405.8	1419.9 ± 219.5	0.68	0.50
Attend Fear vs. Shapes	1337.9 ± 371.9	1285.7 ± 218.5	0.63	0.53
Attend Happy vs. Shapes	1303.7 ± 340.4	1231.0 ± 207.4	0.96	0.34
Attend Shapes vs. Angry (distractor)	1021.1 ± 273.5	957.8 ± 122.4	1.11	0.27
Attend Shapes vs. Fear (distractor)	985.4 ± 239.9	946.2 ± 144.4	0.74	0.47
Attend Shapes vs. Happy (distractor)	975.6 ± 236.4	944.3 ± 157.7	0.58	0.57

### Behavioral data: match shapes

Regarding accuracy, there were no main effects for (distractor) Emotion or Group and no evidence of a (distractor) Emotion x Group interaction (all *p*_*s*_ > 0.05). All participants exhibited similar accuracy when matching shapes alongside angry, fearful, and happy distractor faces. Results for RT were analogous as there were no main effects for (distractor) Emotion or Group and no (distractor) Emotion x Group interaction (all *p*_*s*_ > 0.05). Participants showed comparable RT among each distractor emotion: shapes-angry, shapes-fearful, and shapes-happy (all *p*_*s*_ > 0.05). See Table 2 for behavioral descriptives.

### Functional MRI

**Figure 2:**
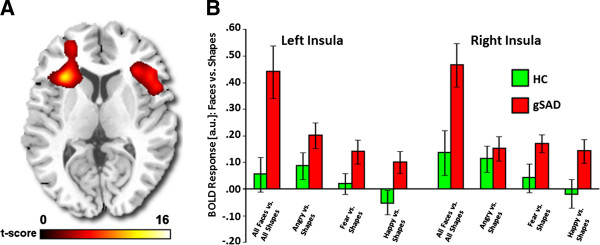
**A priori region of interest: Anterior insula. A**) Voxel-wise main effect of group for the contrast Match Faces > Match Shapes, along with Match Angry > Match Shapes, Match Fear > Match Shapes, and Match Happy > Match Shapes, showing bilateral anterior insula (aINS) displayed on statistical F-map at p < 0.05; cluster size >10 contiguous voxels (family-wise error corrected for multiple comparisons across small volumes of interest). Color scale reflects *F*-score. **B**) Bar graphs depicting extracted parameter estimates of activation from the aINS ROI within each group showing Generalized Social Anxiety Disorder exhibited greater bilateral anterior insula activation than Healthy Controls (p < 0.05).

**Figure 3:**
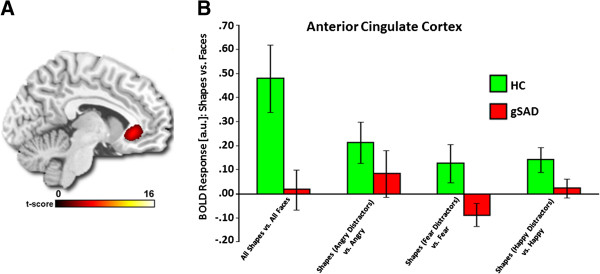
**A priori region of interest: Anterior cingulate cortex. A**) Voxel-wise main effect of group for the contrast Match Shapes > Match Faces, along with Match Shapes (Angry distractor) > Match Angry, Match Shapes (Fear distractor) > Match Fear, and Match Shapes (Happy distractor) > Match Happy, showing anterior cingulate cortex (ACC) displayed on statistical F-map at p < 0.05; cluster size >10 contiguous voxels (family-wise error corrected for multiple comparisons across small volumes of interest). Color scale reflects *F*-score. **B**) Bar graphs depicting extracted parameter estimates of activation from the ACC ROI activation within each group showing Healthy Controls exhibited ACC activation, whereas, Generalized Social Anxiety Disorder showed no ‘activation’.

An ANOVA revealed a main effect of Group for anterior insula and ACC (Table 3). *Post hoc* t-tests showed greater bilateral anterior insula activity for gSAD versus HC [left (−32, 22, 8), F-score = 12.02, volume = 560 mm^3^, _svc_corrected *p* < 0.01; right (32, 26, 4), F-score = 8.03, volume = 440 mm^3^, _svc_corrected *p* < 0.03] when attending to emotional faces (vs. shapes) (Figure 2). Conversely, gSAD showed less activation in left rostral ACC (rACC) compared to HC [(−6, 30, -6), F-score = 6.72, volume = 368 mm^3^, _svc_corrected *p* < 0.05] when attending to shapes (vs. emotional faces) (Figure 3). The main effect of Emotion and the Group x Emotion interaction were non-significant in *a priori* regions.

**Table 3:** Whole-brain voxel-wise analysis of variance

**Region**	**MNI Coordinates**			**Volume**	***F *statistic**
Main Effect of Group					
**Anterior insula**	**-32**	**22**	**8**	**560**	**12.02**
	**32**	**26**	**4**	**440**	**8.03**
**Anterior cingulate cortex**	**-6**	**30**	**-6**	**368**	**6.72**
Middle occipital gyrus	-30	-98	-4	104	16.03
	28	-86	4	248	13.44
Supramarginal gyrus	-64	-42	32	224	14.15
Supplementary motor area	8	-16	52	128	12.43
Main Effect of Emotion					
Hippocampus	-34	-14	-12	104	9.30
Inferior frontal operculum	-36	10	26	112	8.73
Group x Emotion Interaction					
Superior frontal gyrus	-18	32	54	1080	12.01
Caudate	16	-16	24	184	9.91
	22	0	24	152	8.72
Middle frontal gyrus	40	-2	62	104	8.48

*A priori* areas within the atlas-based anatomical regions of interest are shown in bold.

MNI, Montreal Neurological Institute.

### Correlational analyses

Extent of anterior insula (aINS) reactivity in gSAD corresponded with the intensity of social anxiety symptoms (LSAS-total score) though significance was evident in the left aINS (r = 0.32, *p <* 0.04, one-tailed) but not right aINS (r = 0.20, *p =* 0.1, one-tailed). No correlations emerged for aINS reactivity and general anxiety (i.e., trait anxiety; [64] or depression level [62] (all *p*_*s*_ > 0.05) in the gSAD group.

**Figure 4:**
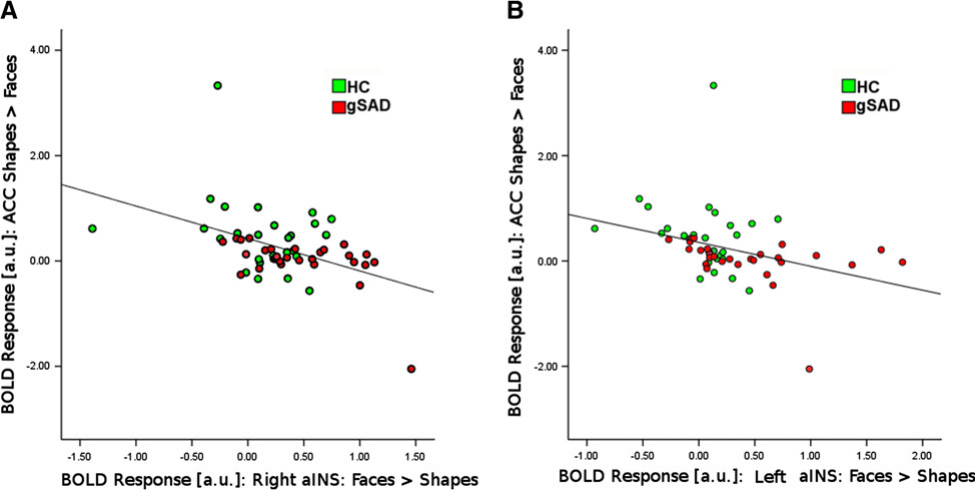
**Correlation between extent of activation from the anterior cingulate cortex (ACC) ROI and extent of activation in A) right anterior insula and B) left anterior insula ROIs across all subjects.** Generalized Social Anxiety Disorder subjects are coded in red; Healthy Controls are coded in green.

We observed a negative relationship between both left aINS and rostral ACC (r = −0.35, *p <* 0.009, two-tailed) and right aINS and rostral ACC (r = −0.45, *p <* 0.001, two-tailed) (Figure 4). Of note, these correlations remained significant even after the removal of the HC and gSAD outliers.

## Discussion

In this functional MRI study, we examined amygdala, anterior insula (aINS), and anterior cingulate cortex (ACC) activation in patients with generalized social anxiety disorder (gSAD) and demographically matched healthy controls (HC) with a novel “Emotional Faces Shifting Attention Task” (EFSAT). The paradigm is an adaptation of widely used emotional faces matching tasks known to evoke exaggerated amygdala and/or insula reactivity in gSAD [17,18]. By spatially combining the traditionally separate faces-only and shapes-only image trials into one trial within one field of view, attention was modulated by having it directed towards or away from emotional faces in order to complete the matching task.

The primary objective in this study was to evaluate attentional modulation with EFSAT in patients with gSAD. When attending to emotional content (Match Faces > Match Shapes), we hypothesized individuals with gSAD relative to HC would exhibit exaggerated amygdala and aINS reactivity particularly to threat expressions (angry, fearful). Our prediction was partially supported. Though no group effects emerged for amygdala, participants with gSAD compared to HC showed bilateral aINS hyper-reactivity when attending to faces, regardless of emotional valence. We also expected a positive correlation between subcortical reactivity and symptom intensity in gSAD. In support, greater left aINS reactivity to emotional faces corresponded with greater symptom severity in gSAD but not with general anxiety or depression level; however, results were weak (i.e., only revealed with one-tailed t-test). Nevertheless, overall findings substantiate the proposal that interoception, a function of aINS that aids in bringing bodily state to awareness [30,31], underlies anxiety by means of negative beliefs that facilitate, or are the result of, exaggerated interoceptive signals [28,29].

When attending away from emotional faces (Match Shapes > Match Faces), we hypothesized gSAD relative to HC would exhibit reduced ACC engagement and that deficient ACC response would be most pronounced in the presence of distracting threat faces. Evidence of enhanced rostral ACC in our HC group is consistent with its role in regulatory control [40,46,65,66]. Specifically, its recruitment when attention was shifted to shapes indicates it served a gating function in modulating the processing of face distractors. We did not find evidence of group effects for dorsal ACC, another region involved in the inhibition of emotional processing [45,66] suggesting EFSAT may not tap into regulatory regions associated with conflict resolution or stimulus appraisal [66]. Again, our hypothesis was partially supported in that gSAD showed a lack of rostral ACC response; however, there was no interaction with the emotional valence of task-irrelevant faces. To our knowledge, the only other study of implicit attentional control in gSAD comprised an event-related emotional conflict task, an emotional counting Stroop paradigm. Results showed patients with gSAD or generalized anxiety disorder lacked dorsal ACC recruitment to congruent and incongruent negative pictures compared to healthy volunteers [49]. Methodological differences do not premit direct comparison with our study, however, evidence of deficient ACC activation during conflict resolution or sustained goal-directed attention suggests impoverished ACC plays a role in excessive attention to emotional information in gSAD and potentially other disorders of emotion dysregulation.

Across participants, we hypothesized a negative relationship between ACC response during attentional control and subcortical (amygdala and/or aINS) reactivity to emotional faces. The prediction was supported, but only in aINS and not amygdala. In a previous study, we showed greater aINS reactivity to threat faces in gSAD than controls, with reduced functional connectivity between aINS and dorsal ACC activity in gSAD indicating exaggerated aINS reactivity was due to a deficiency in ‘top down’ control or appraisal [23]. Here, we observe a negative aINS-ACC relationship across subjects based on direct and indirect emotion processing, respectively, along with evidence of enhanced rostral ACC and reduced bilateral aINS activation in the HC group. Given that the ACC is thought to interact with and/or modulate aINS response to salient signals, we interpret that individuals who exhibit deficient ACC engagement when attentional control is required are those who are likely to exhibit enhanced aINS reactivity when their attention is directed towards emotional stimuli.

## Conclusions

Findings should be considered in the context of several important limitations. Due to the lack of a baseline (e.g., fixation) condition, findings cannot be interpreted in relation to a change from a non-cognitive and non-emotional task. The lack of ‘neutral’ target expressions does not permit dissociation between face and emotion-processing influences. Additionally, the paradigm failed to elicit differential amygdala response between gSAD and controls and insula hyper-reactivity in gSAD was not specific to threat. Together, the task may, in these aspects, not be as sensitive as traditional perceptual matching paradigms. Interestingly, subjects with gSAD have been shown to judge happy faces as less approachable than controls [67]. Although we did not measure potential interpretation bias, we cannot rule out the possibility that insula activation in gSAD involved a less positive judgment of happy faces. Lastly, the gSAD group exhibited greater levels of depression and general anxiety (i.e., trait anxiety) than controls; therefore, we cannot rule out potential influences of depression and general anxiety on results.

Despite limitations, these findings suggest a simple, volitional shift in attention to and away from emotional faces differentiates patients with gSAD from healthy controls in terms of engagement of regions integral to emotion processing and attentional control. During direct and indirect processing of emotional faces, gSAD patients exhibit exaggerated insula reactivity and deficient ACC recruitment, respectively, and different valences, both positive and negative, of socio-emotional information.

## Competing interests

The authors declare that they have no competing interests.

## Authors’ contributions

HK conceived and designed the study, conducted data analyses and interpretation, and wrote the manuscript. DP and DAF conducted data analyses and interpretation, and participated in drafting the manuscript. MA helped design the study, collected the data, and participated in drafting the manuscript. KLP conceived and designed the study, conducted data interpretation, and participated in drafting the manuscript. All authors read and approved the final manuscript.
